# Thyroid abscess associated with thyrotoxicosis caused by *Yersinia enterocolitica* subsp. *palearctica* in a patient with follicular adenoma of the thyroid gland

**DOI:** 10.1186/s12879-024-08974-1

**Published:** 2024-01-08

**Authors:** Takehiro Hashimoto, Takaaki Yahiro, Sonoka Takakura, Sakirul Khan, Kazunori Kimitsuki, Kazufumi Hiramatsu, Akira Nishizono

**Affiliations:** 1 Infection Control Center, Oita University Hospital, Oita, Japan; 2 Department of Microbiology, Oita University Faculty of Medicine, Oita, Japan; 3 Department of Advanced Medical Sciences, Oita University Faculty of Medicine, Oita, Japan; 4 Research Center for Global and Local Infectious Diseases, Oita, Japan; 5 Department of Otolaryngology and Head & Neck Surgery, Oita University Faculty of Medicine, Oita, Japan

**Keywords:** *Yersinia enterocolitica*, Thyroid abscess, Bacteremia

## Abstract

**Background:**

*Yersinia enterocolitica* is a gram-negative zoonotic bacterial pathogen that is typically transmitted via the fecal-oral route. The most common clinical manifestation of a *Y. enterocolitica* infection is self-limited gastroenteritis. Although various extraintestinal manifestations of *Y. enterocolitica* infection have been reported, there are no reports of thyroid abscesses.

**Case presentation:**

An 89-year-old Japanese man with follicular adenoma of the left thyroid gland was admitted to our hospital with a 2-day history of fever and left neck pain. Laboratory tests revealed low levels of thyroid stimulating hormone and elevated levels of free thyroxine 4. Contrast-enhanced computed tomography showed low-attenuation areas with peripheral enhancement in the left thyroid gland. He was diagnosed with thyroid abscess and thyrotoxicosis, and treatment with intravenous piperacillin-tazobactam was initiated after collecting blood, drainage fluid, and stool samples. The isolated Gram-negative rod bacteria from blood and drainage fluid cultures was confirmed to be *Y. enterocolitica*. He was diagnosed with thyroid abscess and thyrotoxicosis due to be *Y. enterocolitica* subsp. *palearctica*. The piperacillin-tazobactam was replaced with levofloxacin.

**Conclusion:**

We report a novel case of a thyroid abscess associated with thyrotoxicosis caused by *Y. enterocolitica* subsp. *palearctica* in a patient with a follicular thyroid adenoma.

## Background

*Yersinia enterocolitica* is a gram-negative zoonotic bacterial pathogen that is typically transmitted via the fecal-oral route [1]. The most common clinical manifestation of a *Y. enterocolitica* infection is self-limited gastroenteritis [2]. Although various extraintestinal manifestations of *Y. enterocolitica* infection have been reported [3], there are no reports of thyroid abscesses. The thyroid gland is rarely infected due to its fibrous capsule, lymphatic drainage, abundant vascularity, and high concentrations of iodine. However, abnormal thyroid anatomy, such as nodular goiters, adenomas, and cysts, can predispose patients to thyroid abscesses. The common pathogens isolated in thyroid abscesses are *Staphylococcus aureus* and *Streptococcus pyogenes*. *Acinetobacter calcoaceticus*, *Eikenella corrodens*, *Escherichia coli*, fungal pathogen, *Helicobacter cinaedi*, *Haemophilus influenzae*, *Klebsiella pneumoniae*, *Mycobacterium tuberculosis*, *Pasteurella multocida,* and *Salmonella* spp. have also been reported as other less common pathogens [4–9]. We report a case of a thyroid abscess associated with thyrotoxicosis caused by *Y. enterocolitica* subsp. *palearctica* in a patient with follicular adenoma of the thyroid gland.

## Case presentation

**Fig. 1:**
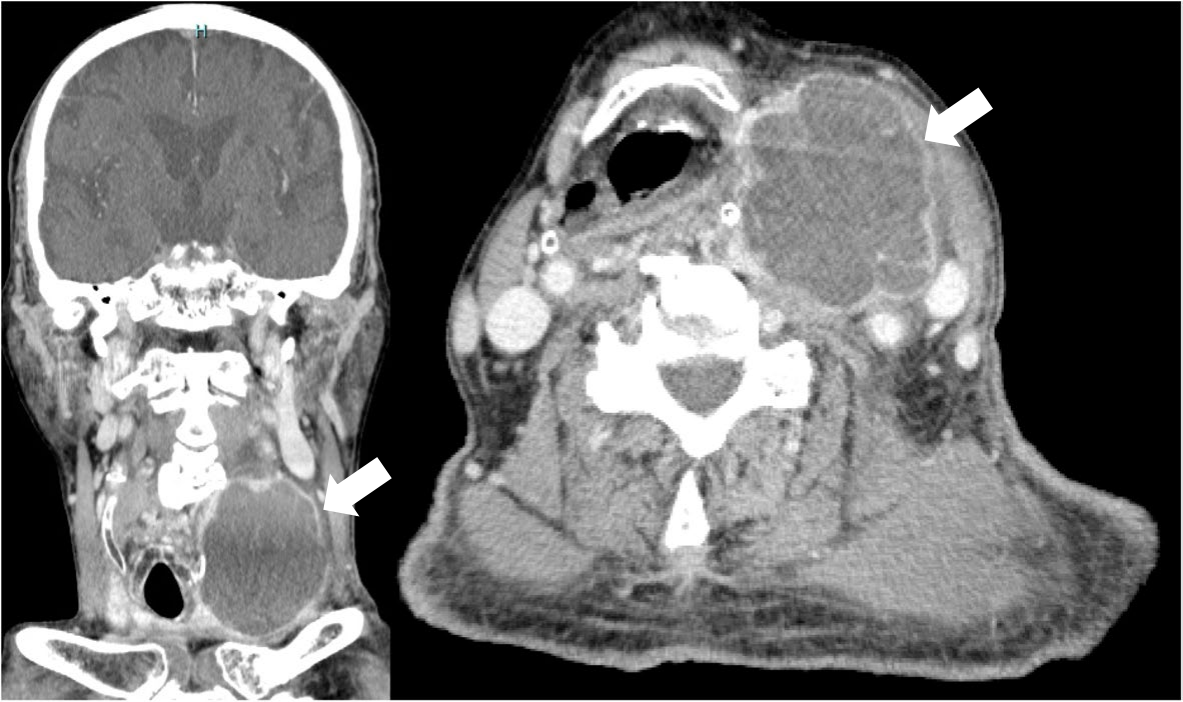
An enhanced computed tomography showing the rim-enhancing fluid collection in the left thyroid grand on axial and sagittal views (white arrow)

**Fig. 2:**
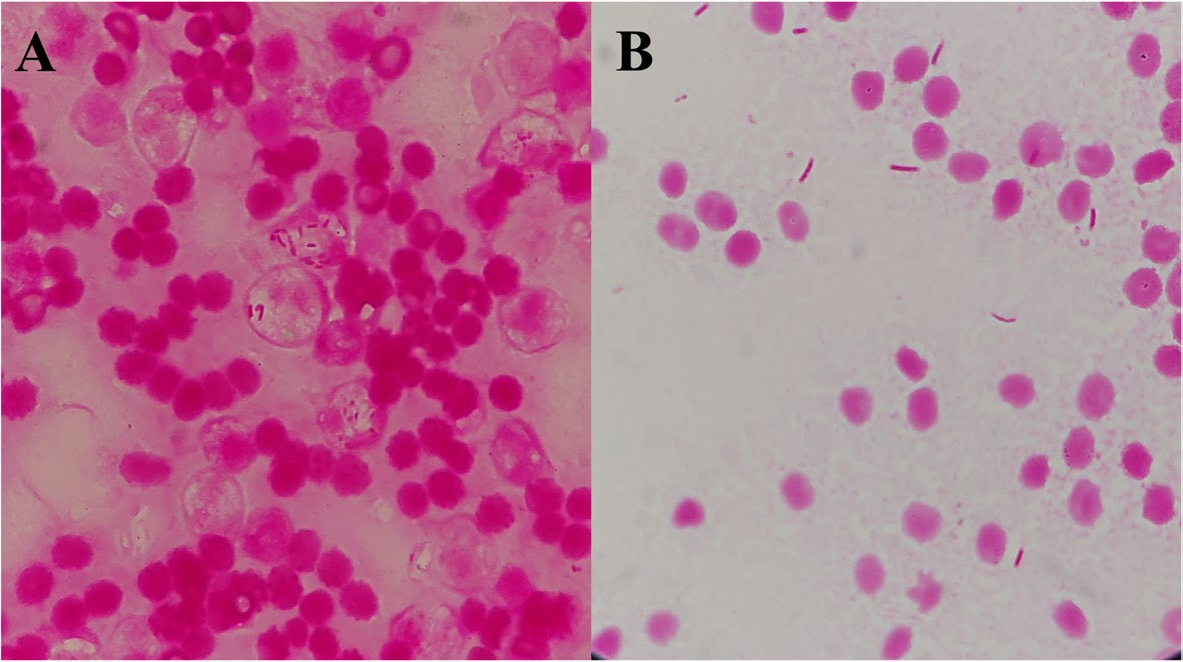
**a** Drainage fluid culture test detecting Gram-negative rod bacteria (Gram stain, magnification 1000 ×). **b** A blood culture test detecting Gram-negative rod bacteria (Gram stain, magnification 1000 ×)

An 89-year-old Japanese man with a follicular adenoma of the left thyroid gland was admitted to the Oita University Hospital (Oita, Japan) with a 2-day history of fever and left neck pain. The patient was diagnosed with the adenoma, approximately three years prior to admission, and his thyroid function was normal five months prior. The patient had a history of congestive heart failure and chronic renal disease. On admission, the patient’s vital signs were as follows: body temperature 38.4 °C; blood pressure, 103/53 mmHg; pulse rate, 89 beats/min; and respiratory rate, 22 beats/min. Physical examination revealed swelling and pain on the left side of the neck. Laboratory tests revealed an elevated white blood cell count (17,520/μL), elevated levels of C-reactive protein (26.5 mg/dL) and free thyroxine 4 (FT4; 1.72 ng/dL), low levels of thyroid-stimulating hormone (TSH; 0.062 μIU/mL) and iron (18 μg/dL), low unsaturated iron-binding capacity (101 μg/dL), low transferrin saturation (15%), and a high B-type natriuretic peptide level (1200 pg/mL). Contrast-enhanced computed tomography showed low-attenuation areas with peripheral enhancement in the left thyroid gland (58 mm × 59 mm) (Fig. 1). The patient was diagnosed with thyroid abscess and thyrotoxicosis. Incision and drainage were subsequently performed, and piperacillin-tazobactam administration was initiated after collecting blood, drainage fluid, and stool samples. On day 2, *Y. enterocolitica* was identified in blood and drainage fluid cultures by matrix-assisted laser desorption/ionization time-of-flight mass spectrometry (MALDI-TOF MS; Bruker Daltonics, Billerica, MA, USA) (score value, 2.127) (Fig. 2). The minimum inhibitory concentrations of the strain, as determined using a dry plate (Eiken, Tokyo, Japan) for the broth microdilution method and analyses by an image analyzer (Koden IA40MIC-i; Koden, Tokyo, Japan), were as follows: ampicillin, 32 μg/mL; ampicillin-sulbactam, 16 μg/mL; cefmetazole, 32 μg/mL; cefotaxime, 4 μg/mL; piperacillin-tazobactam, < 1 μg/mL; meropenem, < 0.12 μg/mL; gentamicin, < 0.25 μg/mL; ciprofloxacin, < 0.5 μg/mL; and levofloxacin, < 0.25 μg/mL. The patient’s stool culture was negative on day five, and they showed defervescence on day seven. The treatment regimen was changed to levofloxacin on day 18, and continued for a total of 42 days. Wound closure was performed on day 31, and the patient was discharged from the hospital on day 35. On day 37, thyroid function was normal (TSH level, 1.34 μIU/mL; FT4 level, 1.5 ng/dL). The *Y. enterocolitica* isolate was further characterized using an O-antigen serotyping scheme, biotyping (esculin, lipase, indole, xylose, and trehalose tests), and 16S ribosomal RNA gene sequencing to evaluate the pathogenicity. The O-antigen serotyping test is performed by slide agglutination using commercial antisera O:3, O:5, O:8, and O:9 for *Y. enterocolitica* (Denka Seiken, Tokyo, Japan). Biotyping test revealed esculin (negative), lipase (negative), indole (positive), xylose (positive) and trehalose (positive). DNA was extracted using the DNA extraction method with the Cica Geneus DNA extraction kit (Kanto Chemical, Tokyo, Japan). 16S ribosomal RNA gene sequencing was performed using the primers 518F (5′-CCAGCAGCCGCGGTAATAC-3′) and 800R (5′- TACCAGGGTATCTAATCC − 3′). Nucleotide sequencing was performed using a BigDye Terminator v3.1 Cycle Sequencing kit and a SeqStudio Genetic Analyzer (Applied Biosystems Inc., Foster City, California, USA). A basic local alignment search tool for the query sequence resulted in the final identification of the bacterium as *Y. enterocolitica* subsp. *palearctica*, which had a 97.8% 16S ribosomal RNA gene sequence similarity (1343 of 1373 nucleotides) to *Y. enterocolitica* subsp. *palearctica* strain (GenBank accession numbers: CP002246.1). The final diagnosis was thyroid abscess with thyrotoxicosis and bacteremia caused by *Y. enterocolitica* subsp. *palearctica* belonging to the serogroup O:9 (biotype 2).

## Discussion and conclusions

We describe the first case of a thyroid abscess associated with thyrotoxicosis due to *Y. enterocolitica* subsp. *palearctica* belonging to the serogroup O:9 (biotype 2). *Y. enterocolitica* is a gram-negative bacterium that has more than 70 serotypes, six biotypes, and two subspecies, *enterocolitica* and *palearctica* [10]. The biotypes can be divided into three groups based on pathogenicity: biotype 1B, high virulence; biotypes 2–5, low virulence; and biotype 1A, nonvirulent [10]. Although the most common clinical manifestation of a *Y. enterocolitica* infection is self-limited gastroenteritis, highly virulent strains can cause extraintestinal manifestations, such as thyrotoxicosis, endocarditis, and meningitis [11]. Although *Y. enterocolitica* O:9 is considered weakly pathogenic, under conditions of iron overload it may become more pathogenic [11]. *Y. enterocolitica* bacteremia has been reported in immunosuppressed individuals and in those with iron overload [3]. Most cases of *Y. enterocolitica* O:9 bacteremia are associated with transfusion [3]; however, a few cases have been reported in patients without iron overload [12]. The thyroid gland is rarely infected due to its fibrous capsule, lymphatic drainage, abundant vascularity, and high concentrations of iodine. However, abnormal thyroid anatomy, such as nodular goiters, adenomas, and cysts, can predispose patients to thyroid abscesses, of which *Staphylococcus aureus* and *Streptococcus pyogenes* are the most common isolates [13]. In the present case, although the stool culture was negative, the patient had intestinal wall edema and reduced intestinal perfusion caused by congestive heart failure. This may have caused bacterial translocation and bacteremia, leading to the thyroid abscess. The patient had thyrotoxicosis upon admission. Thyrotoxicosis is a condition in which the thyroid gland produces excessive amounts of thyroid hormone. It is rarely caused by the destruction of the thyroid gland due to bacterial invasion for acute suppurative thyroiditis and thyroid abscesses [14, 15]. *Escherichia coli*, *Haemophilus influenzae*, *Helicobacter cinaedi*, *Pasteurella multocida,* and *Staphylococcus aureus* have been reported as causative organisms of thyrotoxicosis due to acute suppurative thyroiditis and thyroid abscesses [6–9, 16]. In addition, *Y. enterocolitica* infection has been reported to cause thyroid diseases such as Graves’ disease because antibodies against *Y. enterocolitica* resemble the TSH binding site [17]. In our case, thyroid function returned to normal after antibiotic treatment without antithyroid drugs, and thus, we concluded that the thyrotoxicosis was caused by the thyroid abscess.

Here, we report a novel case of a thyroid abscess associated with thyrotoxicosis caused by *Y. enterocolitica*. Thyrotoxicosis can be caused not only by direct infection of the thyroid gland but also by thyroid disease caused by antibodies against *Y. enterocolitica*. Therefore, if the clinical symptoms associated with *Y. enterocolitica* infection do not improve after appropriate antibiotic treatment, clinicians should consider thyroid diseases related to *Y. enterocolitica* infection and monitor thyroid function.

## Data Availability

All relevant data are within the manuscript.

## Abbreviations

*Y. enterocolitica*: *Yersinia enterocolitica*
TSH: thyroid-stimulating hormone
FT4: free thyroxine 4
